# Bleeding complications after central line insertions and the relevance of pre-procedure coagulation tests and blood component therapy

**DOI:** 10.1186/cc12296

**Published:** 2013-03-19

**Authors:** TK Kander, US Schött

**Affiliations:** 1 Lund University, Lund, Sweden

## Introduction

Bleeding complications in association with insertion of central venous catheters are reported in 0.5 to 1.6% of cases, are rarely fatal, but sometimes require intervention. The aim of this study was to map pre-procedural variables at the insertion of a central venous catheter and to investigate whether any independent variable could be identified as an independent risk factor for bleeding complications associated with insertion of central venous catheters.

## Methods

In this retrospective study, we investigated 1,737 consecutive insertions of central venous catheters on 1,444 patients in a large university hospital during 2009 to 2010. Pre-procedural coagulation status, blood component use, type of catheter, insertion site, and complications during insertion were recorded and compared with bleeding complications documented in electronic charts.

## Results

No serious bleeding complications were recorded in connection with the insertions of central venous catheters. Sixteen out of 1,769 (0.9%) insertions caused grade 2 bleeding, being defined as a bleeding requiring prolonged compression at the insertion site. Insertion of a large-bore central dialysis catheter was found to be an independent risk factor for bleeding complications. Neither conventional coagulation tests nor accidental arterial puncture or the number of needle passes could predict bleeding complications in this study.

## Conclusion

This retrospective study shows that serious bleeding complications in association with central line insertion are uncommon and that insertion of dialysis catheter is an independent risk factor for mild bleeding complications. Coagulopathy, measured with conventional coagulation tests, is not an independent risk factor for bleeding complication at insertion of central venous catheters.

